# Recent case exposure and perceived preparedness for responding to sexual violence in school health services: a cross-sectional survey of school nurses in Japan

**DOI:** 10.1186/s12913-026-15027-7

**Published:** 2026-06-27

**Authors:** Koichi Furuhashi

**Affiliations:** Department of Psychiatry, School of Medicine, Fujita Health University, 1-98 Dengakugakubo, Kutsukake-cho, Toyoake, Aichi 470-1192 Japan

**Keywords:** School nurses, Sexual violence, Perceived preparedness, Interagency collaboration, School health services, Cross-sectional study

## Abstract

**Background:**

School nurses play a key role in responding to sexual violence in school settings. However, it remains unclear whether recent case exposure is associated with greater awareness of institutional response systems and confidence in recognizing sexually inappropriate behaviors.

**Methods:**

We conducted a cross-sectional survey of school nurses in a large Japanese municipality. The primary exposure was self-reported involvement in sexual violence-related cases within the past year (yes/no). The primary outcome was perceived awareness of institutional response procedures and interagency collaboration systems, and the secondary outcome was perceived confidence in recognizing sexually inappropriate behaviors, each measured using a 5-point Likert scale. Group differences were examined using the Mann–Whitney U test.

**Results:**

Among 111 respondents, 50 (45.0%) reported recent exposure to a sexual violence-related case. Recent exposure was not associated with greater perceived awareness of institutional response systems (median 3 [IQR 2–4] vs. 3 [IQR 3–4], *p* = 0.336) or higher perceived confidence in recognizing sexually inappropriate behaviors (median 3 [IQR 3–4] in both groups, *p* = 0.877).

**Conclusions:**

In this sample of Japanese school nurses, recent exposure to sexual violence-related cases was not associated with greater perceived awareness of institutional response systems or higher perceived confidence in recognizing sexually inappropriate behaviors. Because cumulative prior experience, training history, and institutional context were not assessed, these findings should be interpreted cautiously. Future research should examine how professional experience, training opportunities, and organizational factors interact to influence preparedness and response practices related to sexual violence in school settings.

**Supplementary Information:**

The online version contains supplementary material available at https://doi.org/10.1186/s12913-026-15027-7.

## Background

Child and adolescent sexual violence is widely recognized as a major public health concern. A recent global meta-analysis encompassing 165 national studies across 80 countries estimated pooled lifetime prevalence rates of 8.7% for contact sexual violence and 6.1% for completed forced sexual intercourse [1]. Beyond its prevalence, sexual violence during childhood and adolescence is associated with substantial population-level mental health burden, as demonstrated in longitudinal and population-based research [2]. In response to this evidence, international public health organizations have identified the prevention and early identification of sexual violence against children and adolescents as a public health priority, emphasizing the need for effective detection and response mechanisms across community settings [3]. Because children and adolescents spend substantial time within school environments, educational settings may play an important role in the early recognition of sexual violence-related concerns among children and adolescents.

Schools constitute a critical context for the early identification of child and adolescent sexual violence due to their sustained and routine contact with children across developmental stages. Children spend substantial time within educational settings, and school personnel may serve as accessible first-line contacts when maltreatment or sexual violence-related concerns arise [4, 5]. School nurses occupy a unique position within school settings as accessible health professionals who may serve as initial points of contact when health or safety concerns, including sexual violence, arise. Previous empirical studies and integrative reviews have highlighted the potential role of school nurses in recognizing and responding to child maltreatment, including sexual abuse [4, 5].

In Japan, school nurses (yōgo teachers) are responsible for health management, student counseling, and coordination with external agencies such as child guidance centers and medical institutions. As hybrid professionals working across educational and health-related domains, they may play an important role in recognizing and responding to sexual violence-related concerns in school settings. In addition, no nationally standardized training curriculum specifically focused on child sexual abuse (CSA) or sexual violence-related response for school nurses was identified in the available literature and policy documents. Training opportunities and practical experience related to safeguarding and sexual violence-related concerns may therefore vary across institutional and regional contexts. Because much of the existing literature regarding school nurses and responses to sexual violence-related concerns, including CSA, originates from Western contexts [4, 5], it remains unclear whether previous findings concerning professional experience, confidence, and safeguarding practices are transferable to school health systems with different organizational and professional structures.

At the same time, the literature consistently documents challenges faced by school nurses in safeguarding roles, including limited training opportunities, time constraints, emotional burden, and uncertainty regarding professional roles and responsibilities [4, 5]. As a result, proactive identification and structured screening for CSA may be limited in some school contexts. Qualitative evidence suggests that school nurses may hesitate to raise concerns about CSA and may find it difficult to ask direct or sensitive questions [6].

Despite growing recognition of the potential role of school nurses in responding to CSA, empirical evidence regarding factors that facilitate effective identification and response remains limited. In particular, the relationship between professional experience and perceived confidence or self-efficacy in recognizing and responding to sexual violence has received relatively little attention in the context of recent case exposure within school settings.

Survey-based studies indicate that school nurses frequently report concerns about having overlooked cases of CSA. In one cross-sectional study, self-efficacy and perceived professional responsibility predicted intention to screen, whereas knowledge of clear and severe indicators of abuse predicted intention to report suspected cases [7]. Although some studies have reported differences in recognition and reporting practices according to years of professional experience [8], this evidence is largely derived from Western contexts. Consequently, important gaps remain in understanding how recent exposure to sexual violence-related cases relates to school nurses’ perceived confidence and awareness of response systems in non-Western settings.

Against this background, this study aimed to examine the association between recent exposure to sexual violence-related cases and school nurses’ perceived awareness of institutional response systems, as well as their perceived confidence in recognizing sexually inappropriate behaviors. Using data from a cross-sectional survey of school nurses in Japan, we explored whether exposure within the past year was associated with enhanced recognition of institutional response frameworks or greater perceived confidence, which may provide preliminary insights relevant to school health services in other contexts. Given the limited prior evidence, all analyses were conducted in an exploratory and hypothesis-generating manner.

## Methods

### Participants

This study employed a cross-sectional questionnaire design. Participants were school nurses (yōgo teachers) employed at elementary, junior high, senior high, and special needs schools in a large metropolitan area of Japan.

### Procedure

Data were collected during an annual in-person mental health training workshop for school nurses organized by the municipal board of education. Workshop themes varied annually, and the 2025 workshop focused on child and adolescent sexual violence and related mental health concerns. The author was invited as an external lecturer based on this annual theme selection.

The survey was conducted independently of the educational program and was intended to explore school nurses’ prior experiences and perceptions regarding sexual violence-related cases in school settings. The workshop was not designed as a structured intervention study evaluating changes in preparedness, confidence, or knowledge.

All school nurses attending the workshop were invited to voluntarily complete an anonymous, self-administered survey. According to the municipal board of education, there were 428 school nurses in the municipality at the time of the survey. Of the 287 school nurses invited, 111 provided valid responses via Google Forms, yielding a response rate of 38.7% among workshop attendees.

The survey was open from the date of the workshop (December 13, 2025) until the response deadline (December 31, 2025). Participation was voluntary, and no financial or non-financial incentives were offered. Electronic informed consent was obtained from all participants before survey completion. The survey was available after the workshop and could be completed voluntarily at any time during the study period. This study received ethical approval from the Institutional Review Board of Fujita Health University (approval number HM25-357; November 7, 2025) and was conducted in accordance with the Declaration of Helsinki.

### Instrumentation

The questionnaire was developed by the author based on prior literature concerning school nurses’ roles in child maltreatment and CSA identification, safeguarding practices, and perceived preparedness [4, 5, 7]. Because the study was designed as an exploratory investigation, the questionnaire consisted primarily of brief self-report items intended to assess recent exposure, perceived awareness of institutional response systems, and perceived confidence in recognizing sexually inappropriate behaviors. These items assessed respondents’ experiences with and perceptions of sexual violence-related cases in school settings. All items were developed for this study unless otherwise specified.

Perceived awareness of institutional response frameworks was assessed using a single Likert-scale item from 1 “not aware at all” to 5 “very aware”. This variable was treated as the primary outcome and analyzed as an ordinal variable.

Recent exposure to sexual violence-related cases was assessed as a binary variable (yes/no). Exposure was defined as having been involved in the identification, consultation, or response to at least one sexual violence-related case within the school context during the preceding 12 months.

Perceived confidence in recognizing sexually inappropriate behaviors was measured using a single-item question on a 5-point Likert scale (1 = “not confident at all” to 5 = “very confident”). This item assessed respondents’ perceived confidence in recognizing sexually inappropriate behaviors. This measure was treated as a secondary outcome and analyzed as an ordinal variable.

The full questionnaire is provided in the Supplementary Material.

### Data analysis

Statistical analyses were conducted using EZR version 1.64, a graphical user interface for R and R Commander designed for medical statistics [9].

The primary exposure variable was recent exposure to sexual violence-related cases within the past year (yes/no). The primary outcome was perceived awareness of institutional response frameworks, measured on a 5-point ordinal scale. The secondary outcome was perceived confidence in recognizing sexually inappropriate behaviors. Group differences in ordinal outcomes were examined using the Mann–Whitney U test.

Given the ordinal nature of the outcome measures and the presence of substantial ties, effect size estimates were not calculated. Descriptive statistics were used to summarize participant characteristics. No additional subgroup analyses were conducted due to the exploratory and hypothesis-generating nature of the study.

All analyses were exploratory and hypothesis-generating.

### Use of large language models in the writing process

During the preparation of this work, the author used ChatGPT (OpenAI) to refine English expressions, organize sentence structure, and respond to copyediting comments. After using this tool/service, the author reviewed and edited the content as needed and takes full responsibility for the content of the published article.

## Results

Of the 287 school nurses invited to participate, 111 (38.7%) provided valid responses. Participants’ professional characteristics are presented in Table 1.

Among respondents, 50 (45.0%) reported recent exposure to sexual violence-related cases within the past year, whereas 61 (55.0%) reported no such exposure.

**Table 1:** Characteristics of participating school nurses (*n* = 111)

Characteristic	*n* (%)
School type	
Elementary school	69 (62.2)
Junior high school	37 (33.3)
High school	5 (4.5)
Special needs school	0 (0.0)
Years of experience as a school nurse	
< 1 year	1 (0.9)
1–5 years	19 (17.1)
6–10 years	20 (18.0)
11–20 years	37 (33.3)
≥ 21 years	34 (30.6)
Training related to sexual violence or abuse^a^	
School or board of education-led training	106 (95.5)
Academic institutions or professional societies	15 (13.5)
National or local government	11 (9.9)
Private or other organizations	19 (17.1)
No training experience	2 (1.8)
In-school documentation format for incidents	
Standardized format available	39 (35.1)
No standardized format	53 (47.7)
Not sure	19 (17.1)

Note. Values are presented as numbers (percentage)

^a^Multiple responses were allowed

### Primary outcome

Perceived awareness of in-school response procedures and collaboration with external agencies likewise did not differ significantly between groups (Table 2). The median awareness score was 3 (interquartile range [IQR], 2–4) among respondents without recent exposure and 3 (IQR, 3–4) among those with recent exposure (Mann–Whitney U test, *p* = 0.336).

### Secondary outcome

Perceived confidence in recognizing sexually inappropriate behaviors did not differ significantly between respondents with and without recent exposure to sexual violence-related cases (Table 2). The median confidence score was 3 (IQR, 3–4) in both groups (Mann–Whitney U test, *p* = 0.877).

**Table 2:** Association between recent exposure to sexual violence-related cases and perceived awareness and confidence measures

Outcome measure	No recent exposure (*n* = 61)	recent exposure (*n* = 50)	*p* value
**Primary outcome**			
Perceived awareness of in-school procedures and collaboration with external agencies	3 (2–4)	3 (3–4)	0.336
**Secondary outcome**			
Perceived confidence in recognizing sexually inappropriate behaviors	3 (3–4)	3 (3–4)	0.877

Note. Values are presented as median (interquartile range)

All items were rated on a 5-point Likert scale

Group differences were examined using the Mann–Whitney U test

All analyses were exploratory

## Discussion

### Principal findings

In this cross-sectional survey of school nurses in Japan, recent exposure to sexual violence-related cases was not associated with greater perceived awareness of school-based response manuals or interagency collaboration systems. Likewise, such experience was not associated with higher perceived confidence in recognizing sexually inappropriate behaviors. Collectively, these findings suggest that recent case exposure alone may not necessarily correspond to differences in perceived awareness or confidence among school nurses within school settings.

### Comparison with prior literature

Previous literature has similarly suggested that school nurses’ preparedness and engagement in safeguarding practices may be shaped by multiple factors, including access to training, organizational support, and clarity of professional roles [4, 5, 7]. Within this context, the absence of a significant association in the present study should not be interpreted as evidence that experiential exposure lacks relevance, but rather as an indication that preparedness and response processes may be more complex than can be captured by recent exposure alone.

The present findings are broadly consistent with prior research documenting challenges faced by school nurses in responding to CSA [5, 6]. Previous studies have shown that school nurses often report limited training opportunities, emotional burden, and uncertainty regarding their safeguarding responsibilities, factors that may influence the consistency and perceived adequacy of responses even when cases are encountered [5, 6].

Evidence from integrative reviews and cross-sectional surveys further suggests that perceived preparedness and engagement in identification, reporting, and response practices may be shaped by multiple factors, including access to training, clarity of professional roles, and institutional support structures, rather than by case exposure alone [4, 7]. Furthermore, prior research has demonstrated variability in recognition and reporting practices according to years of professional experience, indicating that experiential exposure may interact with career stage and cumulative training experiences in complex ways [8].

Within this broader literature, the absence of a direct association observed in the present study may reflect the multifactorial and context-dependent nature of preparedness and response processes related to sexual violence in school settings.

### Interpretation

The lack of association between recent exposure to sexual violence-related cases and perceived confidence or system awareness may reflect multiple factors. In the present study, exposure was defined as involvement in sexual violence-related cases within the preceding 12 months and did not account for cumulative prior professional experiences. Consequently, respondents categorized as having no recent exposure may nevertheless have had substantial prior experience with similar cases, which may have reduced observable between-group differences. In addition, preparedness and confidence in responding to sexual violence-related cases are likely influenced by a complex interplay of factors, including prior training experiences, institutional context, and professional role expectations.

These findings may suggest that recent case exposure alone does not necessarily correspond to differences in perceived awareness or confidence within school settings, where preparedness may develop through cumulative experiences that vary across institutional and professional contexts.

### Limitations

Several limitations should be considered when interpreting these findings. First, the cross-sectional design precludes causal inference regarding the relationship between experience, confidence, and system awareness. In addition, exposure was defined as involvement in sexual violence-related cases within the preceding 12 months and did not account for cumulative prior professional experiences, which may have reduced observable differences between groups. Second, subgroup analyses by school type or years of professional experience were not conducted because the limited sample size would have resulted in small cell sizes and unstable estimates, which were considered beyond the scope of this exploratory investigation. Demographic characteristics such as age, gender, and racial or ethnic background were not collected. Although years of professional experience were assessed, unmeasured demographic and professional factors may also have influenced respondents’ perceptions and experiences. Participants were recruited from a municipal training workshop that school nurses in the area were generally expected to attend. Of the 428 school nurses in the municipality, 287 attended the workshop and 111 provided valid responses. Although this setting allowed access to a broad group of school nurses within the municipality, the response rate may introduce response bias. Because the survey was anonymous and voluntary, individual-level information regarding non-respondents was unavailable, and direct comparisons between respondents and non-respondents could not be performed.

In addition, all measures relied on self-report, which may be subject to recall or social desirability bias. The use of single-item measures for perceived confidence and system awareness may also limit measurement precision. Despite these limitations, the study provides preliminary evidence that can inform hypothesis generation and guide future research.

### Implications for future research and school health practice

The present findings suggest that preparedness related to sexual violence-related concerns in school settings may be influenced by multiple factors beyond recent case exposure alone. Because cumulative professional experience, prior training history, and institutional context were not assessed in the present study, the absence of a significant association should be interpreted cautiously.

Future research should examine how organizational context, professional experience, and training opportunities interact to shape preparedness and response practices among school nurses. In particular, longitudinal and intervention-based studies may help clarify factors associated with effective school-based responses to sexual violence-related concerns.

Given the hybrid educational and health-related roles of Japanese school nurses, further investigation of how school health systems support coordination, consultation, and response processes may also be warranted.

## Conclusions

In this cross-sectional survey of school nurses in Japan, recent exposure to sexual violence-related cases within the past 12 months was not associated with greater awareness of institutional response systems or higher perceived confidence in recognizing sexually inappropriate behaviors.

Because cumulative prior exposure, training history, and institutional context were not assessed, the findings should be interpreted cautiously. The absence of a significant association may reflect the complex and heterogeneous factors that shape preparedness and responses to sexual violence-related concerns in school settings.

Future research should examine how professional experience, training opportunities, and organizational factors interact to influence preparedness and response practices among school nurses. Longitudinal and intervention-based studies may help clarify factors associated with effective school-based responses to sexual violence.

## Supplementary Information

Below is the link to the electronic supplementary material.

Supplementary Material 1: Questionnaire Used in the Survey of School Nurses in Japan.

## Data Availability

The datasets generated and/or analyzed during the current study are not publicly available due to the risk of participant identification in a single-municipality sample but are available from the corresponding author on reasonable request, subject to institutional and ethical approval.

## Abbreviations

CSA: Childhood sexual abuse
IQR: Interquartile range
