# Supplementary material for: Recent case exposure and perceived preparedness for responding to sexual violence in school health services: a cross-sectional survey of school nurses in Japan
Source: BMC Health Serv Res. 2026 Jun 27;26:1267. doi: 10.1186/s12913-026-15027-7 (PMC13576135; doi:10.1186/s12913-026-15027-7)
Supplement: Supplementary file 1 [file 12913_2026_15027_MOESM1_ESM.docx]

**Supplementary Material 1**

**Questionnaire Used in the Survey of School Nurses in Japan**
(*Translated from the original Japanese questionnaire*)

**Informed Consent**

This survey was conducted to examine school nurses’ recognition of and responses to sexual violence involving children and adolescents.

- Participation was voluntary.
- Responses were anonymous.
- No individual participant could be reasonably identified from the data.
- Data were analyzed statistically and may be presented in academic publications.

Participants provided informed consent electronically before proceeding to the questionnaire.

**Section 1. Basic Information**

**Q1. School Type**

(*Select one*)

- Elementary school
- Junior high school
- Senior high school
- Special needs school

**Q2. Years of Experience as a School Nurse**

(*Select one*)

- Less than 1 year
- 1–5 years
- 6–10 years
- 11–20 years
- 21 years or more

**Q3. Training Related to Sexual Violence or Abuse**

(*Multiple responses allowed*)

- Training led by the school or board of education
- Training led by academic institutions or professional societies
- Training led by the national or local government
- Training led by private or other organizations
- No training experience

**Section 2. Knowledge and Awareness**

**Q4. Understanding the Definition of Sexual Violence**

*To what extent do you feel that you correctly understand the definition of sexual violence?*

Response scale (5-point Likert):

1 = Not at all
2 = Slightly
3 = Somewhat
4 = Mostly
5 = Very much

**Q5. Types of Sexual Violence That May Require a School-Based Response**

(*Multiple responses allowed*)

- Adult–student cases
- Student–student cases (including same-sex cases)
- Teacher or school staff–student cases
- Online cases (e.g., social network services (SNS)-related incidents)

**Q6. Awareness of In-School Manuals and External Collaboration**

*How well do you understand the school’s response manuals and collaboration systems with external agencies (e.g., child guidance centers, police, medical institutions)?*

Response scale (5-point Likert):

1 = Not aware at all
2 = Slightly aware
3 = Moderately aware
4 = Mostly aware
5 = Very aware

(*Used as the secondary outcome measure in the present study*.)

**Section 3. Experience Within the Past Year**

**Q7a. Number of Encounters With Sexual Violence–Related Cases (Past 12 Months), by Relationship Type**

*For each category, select one response*.

Response options: 0/1/2–3/4 or more

- Adult–student
- Student–student
- Teacher or school staff–student

**Q7b. Number of Encounters With Sexual Violence–Related Cases (Past 12 Months), by Modality**

Response options: 0/1/2–3/4 or more

- Face-to-face cases
- Online cases (e.g., SNS)

**Q7c. Experience of Sexual Violence–Related Case in the Past Year**

*Have you encountered at least one sexual violence*–related case in the past 12 months?

- Yes (one or more cases)
- No (no cases)

(*Used as the primary exposure variable in the present study*.)

**Section 4. Response to Cases**

**Q8a. Actions Taken When Encountering a Case**

(*Multiple responses allowed*)

- Interviewed the student(s)
- Shared information within the school (e.g., homeroom teacher, administrators)
- Contacted or consulted guardians
- Consulted the school counselor or a school social worker
- Referred to or consulted external agencies (e.g., child guidance center, police, one-stop support center)
- Prioritized protection of the student(s) (e.g., safety measures, separation)
- Unable to respond adequately
- Other (free text)

**Q8b. Free Description (Optional)**

**Section 5. Recognition of Signs**

**Q9. Confidence in Judging Possible Signs of Sexual Violence**

*If you observe the following signs, how confident are you in making an appropriate judgment?*

Response scales (5-point Likert):

1 = Not confident at all
2 = Slightly confident
3 = Neither confident nor not confident
4 = Somewhat confident
5 = Very confident

Items:

- Unexplained physical symptoms (e.g., abdominal pain, headache)
- School refusal, frequent tardiness, or absenteeism
- Anxiety, panic, or emotional instability
- Self-harm behaviors (e.g., wrist cutting)
- Sexually inappropriate words or behaviors
- Withdrawal or isolation from peers

*(The primary outcome in the present study was perceived confidence in recognizing sexually inappropriate behaviors.)*

**Section 6. Barriers to Reporting and Collaboration**

**Q10. Factors That May Hinder Reporting or Interagency Collaboration**

*(Multiple responses allowed)*

- Lack of sufficient evidence or difficulty establishing facts
- Difficulty responding to or communicating with guardians
- Need to await administrative judgment or approval
- Barriers to contacting external agencies (e.g., child guidance centers, police, medical institutions)
- Previous unsatisfactory responses from external agencies
- Concern about school reputation or impact on stakeholders
- Procedural complexity or lack of time
- Manuals are unclear or not up to date
- Concern about the secondary victimization of students
- Anxiety regarding confidentiality and privacy protection
- Lack of confidence in one’s own knowledge or skills
- Other (free text)
- None

**Q11. Free Description (Optional)**

**Section 7. Peer-to-Peer Cases**

**Q12. Experience of Peer-to-Peer Cases**

*Have you encountered cases of suspected sexual violence or harm between students (including same-sex cases)?*

- Yes
- No
- Not sure

**Q13a. Anticipated Response to Peer-to-Peer Cases**

*(Multiple responses allowed)*

- Ensure the safety of the students involved (the alleged victim and the alleged perpetrator)
- Prevent secondary victimization
- Contact and consult guardians
- Refer or consult medical institutions or one-stop support centers
- Share information and report within the school
- Preserve records and documentation
- Coordinate with school administration and consider a school-level response
- Collaborate with external agencies (e.g., child guidance centers, police)
- Other (free text)

**Q13b. Free Description (Optional)**

**Section 8. Documentation and Privacy**

**Q14. Availability of a Standardized Documentation Format**

For Incidents

- Yes
- No
- Not sure

**Q15a. Measures to Ensure Privacy During Consultation and Record-Keeping**

*(Multiple responses allowed)*

- Use a dedicated interview or counseling room
- Securing a space where other staff cannot observe the interaction
- Adjusting consultation timing to avoid visibility by others
- Limiting accompanying personnel (e.g., teachers, health room staff)
- Minimizing the scope of in-school documentation and information sharing
- Privacy protection is difficult to ensure
- Other (free text)

**Q15b. Free Description (Optional)**

**Section 9. Training Needs**

**Q16. Desired Training Topics**

*Response scale (5-point Likert): Strongly do not want to Strongly want*

- Initial interviews with students
- Education about consent and sexual education
- Responding to guardians
- Practical procedures for reporting and notification
- Development and use of crisis response manuals
- Collaboration with one-stop support centers for victims of sexual violence
- Response to peer-to-peer cases
- Response to online victimization (e.g., SNS-related)

**Q17a. Preferred Training Format**

*(Multiple responses allowed)*

- Lecture-based format
- Case-based discussion format
- Role-play format
- Checklist- or guideline-based learning
- Joint training with administrators or other school staff
- Online training
- Lectures delivered by external experts
- Other (free text)

**Q17b. Free Description (Optional)**
